# Correspondence: is there an association between centre volume and survival or neurological outcomes among out-of-hospital cardiac arrest patients?

**DOI:** 10.1186/s12873-022-00743-0

**Published:** 2022-12-10

**Authors:** Amelia Xin Chun Goh, Andrew Fu Wah Ho

**Affiliations:** 1 Yong Loo Lin School of Medicine, National University of Singapore, 10 Medical Dr, Singapore, 117597 Singapore; 2 Department of Emergency Medicine, Singapore General Hospital, Singapore, Singapore; 3 Pre-Hospital and Emergency Research Centre, Duke-NUS Medical School, Singapore, Singapore

**Keywords:** Sudden cardiac arrest, Heart arrest, Cardiac arrest centre, Volume-outcome relationship, Hospital characteristics, Hospital volume

## Abstract

This commentary discusses the findings of a study by Tsuchida et al. on the effect of annual hospital admissions of out-of-hospital cardiac arrest patients on survival and neurological outcomes in OHCA patients in the context of existing literature on the topic, and the implications on future studies investigating the volume-outcome relationship in cardiac arrest.

This commentary discusses the findings of a study by Tsuchida et al. on the effect of annual hospital admissions of out-of-hospital cardiac arrest (OHCA) patients on survival and neurological outcomes in OHCA patients in the context of existing literature on the topic, and the implications on future studies investigating the volume-outcome relationship in cardiac arrest.

Tsuchida et al. reported the results of a retrospective study which analysed the data of 3632 patients hospitalised for OHCA of cardiac aetiology at 86 hospitals from the Japanese Association for Acute Medicine OHCA registry, a nationwide multihospital prospective database. The main finding of the study was that transport of OHCA patients with prehospital return of spontaneous circulation (ROSC) to a high-volume centre may improve neurological outcomes at 30 days (OR 1.955, 95% confidence interval [CI] 1.033–3.851) [1]. They did not find any significant association between centre volume and survival or neurological outcomes in patients without prehospital ROSC.

The objective of this editorial commentary is to reconcile the conflicting results of current studies regarding the association of centre volume and outcomes in patients with OHCA. The key question we aim to discuss is: Does centre volume alone truly impact outcomes in OHCA patients? Currently, the evidence for high-volume centres remains largely inconclusive. A systematic review by Yeung et al. concluded from pooled data from two studies (*n* = 3673) that care at a cardiac arrest centre (CAC), the definition of which includes ‘high case volume centres’, was associated with increased likelihood of surviving to hospital discharge with favourable neurological outcome compared to other hospitals (OR 2.22 95% CI 1.74–2.84) [2]. As a result, the International Liaison Committee on Resuscitation (ILCOR) guidelines were updated to recommend that adult non-traumatic OHCA cardiac arrest patients be cared for in CACs rather than in non-CACs [3]. While Tsuchida et al.'s findings add to the pool of evidence for high-volume centres, there is still a significant number of studies that have not found a significant association between centre volume and survival or neurological outcomes. For example, a recent systematic review undertaken by the authors of this letter found no association between centre volume and neurological outcomes, although there may be a slight survival benefit [4]. These findings are diametrically opposed to that of Tsuchida et al.'s. A possible reason for this discrepancy, and a major limitation of many studies on centre volume, is hospital characteristics. The additional neurological benefit in patients with prehospital ROSC who were sent to high volume centres could be attributed to the hospitals’ availability of 24/7 access to percutaneous coronary intervention (PCI) or therapeutic temperature management (TTM), or extracorporeal membrane oxygenation (ECMO). These features comprise the bundle of interventions available in CACs, which was shown in a recent systematic review and meta-analysis by Yeo et al. to improve survival and neurological outcomes [5]. Another recent study by Yoon et al. also found that amongst patients who achieved prehospital ROSC, transfer to a CAC was associated with a higher probability of favourable neurological recovery and survival to discharge compared to conveyance to a non-CAC [6]. Unfortunately, as mentioned by Tsuchida et al., limited information was available on the differences in hospital characteristics, thereby precluding the utilization of factors related to hospitals as covariates in their generalized estimating equations [1]. This is a significant limitation as 24/7 PCI and TTM are important confounders of survival and neurological benefit in OHCA patients. While Tsuchida et al. performed a subgroup analysis for patients who were transported to ECMO-capable hospitals, it is important to note that most of the low- and middle-volume hospitals included in their study were also ECMO-capable and there was no significant difference in ECMO-capability amongst the volume tertiles (*p* = 0.061). This may have accounted for the lack of significant association found between hospital volume and survival or neurological outcomes in the subgroup of patients sent to ECMO-capable hospitals. Considering that even the smaller hospitals in this study were well-equipped with invasive and advanced technologies such as ECMO, which is currently not the case worldwide, the organisation of the Japanese territory in question may not be generalizable to the global setting not just in terms of availability of resources but also, different Emergency Medical Services (EMS), resuscitation practises on transport, and hospital organizations. This may have contributed to the difference in results with the recent systematic review [4]. While on the subject of ECMO, studies have also demonstrated the feasibility and benefits of extracorporeal cardiopulmonary resuscitation (ECPR) in increasing survival with good neurological outcomes [7], which indirectly increases the evidence in support of transporting and treating OHCA patients in CACs.

In conclusion, current evidence is insufficient to ascertain if centre volume should be a key factor in deciding where OHCA patients, regardless of patient characteristics, should be transported. Future studies are encouraged to adjust for other components of post-cardiac arrest care, such as 24/7 PCI, TTM and ECMO capability, to determine if centre volume alone is associated with survival or neurological outcomes in OHCA patients.

## Data Availability

Not applicable.

## Abbreviations

CAC: Cardiac arrest centre
CI: Confidence interval
ECPR: Extracorporeal cardiopulmonary resuscitation
ECMO: Extracorporeal membrane oxygenation
EMS: Emergency medical services
ILCOR: International Liaison Committee on Resuscitation
OHCA: Out-of-hospital cardiac arrest
PCI: Percutaneous coronary intervention
ROSC: Return of spontaneous circulation
TTM: Therapeutic temperature management
